# Role for Chromatin Remodeling Factor Chd1 in Learning and Memory

**DOI:** 10.3389/fnmol.2019.00003

**Published:** 2019-01-23

**Authors:** Ines Schoberleitner, Anna Mutti, Anupam Sah, Alexandra Wille, Francisco Gimeno-Valiente, Paolo Piatti, Maria Kharitonova, Luis Torres, Gerardo López-Rodas, Jeffrey J. Liu, Nicolas Singewald, Christoph Schwarzer, Alexandra Lusser

**Affiliations:** ^1^Division of Molecular Biology, Biocenter, Medical University of Innsbruck, Innsbruck, Austria; ^2^Department of Pharmacology, Medical University of Innsbruck, Innsbruck, Austria; ^3^Department of Pharmacology and Toxicology, Institute of Pharmacy and Centre for Molecular Biosciences (CMBI), Leopold-Franzens University of Innsbruck, Innsbruck, Austria; ^4^Institute of Health Research, INCLIVA, and Department of Biochemistry and Molecular Biology, University of Valencia, Valencia, Spain; ^5^Department of Proteomics and Signal Transduction, Max-Planck-Institute of Biochemistry, Munich, Germany

**Keywords:** chromatin, learning, memory, cognition, epigenetics, immediate early genes, hippocampus, gene expression

## Abstract

Precise temporal and spatial regulation of gene expression in the brain is a prerequisite for cognitive processes such as learning and memory. Epigenetic mechanisms that modulate the chromatin structure have emerged as important regulators in this context. While posttranslational modification of histones or the modification of DNA bases have been examined in detail in many studies, the role of ATP-dependent chromatin remodeling factors (ChRFs) in learning- and memory-associated gene regulation has largely remained obscure. Here we present data that implicate the highly conserved chromatin assembly and remodeling factor Chd1 in memory formation and the control of immediate early gene (IEG) response in the hippocampus. We used various paradigms to assess short-and long-term memory in mice bearing a mutated *Chd1* gene that gives rise to an N-terminally truncated protein. Our data demonstrate that the *Chd1* mutation negatively affects long-term object recognition and short- and long-term spatial memory. We found that Chd1 regulates hippocampal expression of the IEG *early growth response 1* (*Egr1*) and *activity-regulated cytoskeleton-associated* (*Arc*) but not *cFos* and *brain derived neurotrophic factor* (*Bdnf*), because the *Chd1*-mutation led to dysregulation of *Egr1* and *Arc* expression in naive mice and in mice analyzed at different stages of object location memory (OLM) testing. Of note, Chd1 likely regulates *Egr1* in a direct manner, because chromatin immunoprecipitation (ChIP) assays revealed enrichment of Chd1 upon stimulation at the *Egr1* genomic locus in the hippocampus and in cultured cells. Together these data support a role for Chd1 as a critical regulator of molecular mechanisms governing memory-related processes, and they show that this function involves the N-terminal serine-rich region of the protein.

## Introduction

Eukaryotic chromatin, which consists of nuclear DNA complexed with the conserved histone proteins H2A, H2B, H3, H4 and H1 as well as many additional architectural proteins, is a highly dynamic structure that changes in response to external and internal cues as well as during developmental and differentiation-related processes. DNA methylation, histone modification and the incorporation of histone variants strongly affect the functional and structural properties of chromatin (Maison and Almouzni, 2004; Piatti et al., 2011; Zeilner et al., 2012). In addition, the activity of ATP-dependent chromatin remodeling factors (ChRFs) that modulate the interaction between histones and DNA causes the loss or gain of nucleosomes, altered rotational and translational positioning of a nucleosome, histone exchange or changes in nucleosomal structure (Lusser and Kadonaga, 2003; Clapier and Cairns, 2009). Thus, ChRFs are critically involved in controlling access to the DNA for protein machineries that act on DNA as their substrate, such as the transcription apparatus. Establishment and maintenance of a certain transcriptional activity state is typically achieved by a close cooperation between transcription factors and cofactors with the machinery that modulates chromatin structure including ChRFs, histone- and DNA-modifying activities. The importance of these epigenetic regulatory mechanisms for cognitive processes has been increasingly recognized in recent years (Sweatt, 2013; Rudenko and Tsai, 2014; López and Wood, 2015; Grigorenko et al., 2016; Iwase et al., 2017; Schmauss, 2017; Gallegos et al., 2018; Wood, 2018).

Learning and memory formation require changes in gene expression programs and new protein synthesis, which in turn results in long-lasting forms of synaptic plasticity, such as long-term potentiation (LTP) and long-term depression (LTD; Howland and Wang, 2008). Epigenetic mechanisms have been proposed to act as a “gating” mechanism coordinating gene expression changes important for learning on one hand, and on the other hand to serve a “stabilizing” role by controlling transcriptional changes important for memory consolidation (Guan et al., 2015). To date, most studies have focused on the roles of DNA methylation, histone acetylation and methylation in cognitive processes (Rudenko and Tsai, 2014; Grigorenko et al., 2016), while very little is known about the contribution of ATP-dependent ChRFs. A notable exception is the nBAF complex with its catalytic subunit Brg1, which belongs to the SWI/SNF subfamily of ChRFs. nBAF was shown to play an important part in memory formation and consolidation (Vogel-Ciernia et al., 2013, 2017; White et al., 2016; Yoo et al., 2017). Recently, a study using heterozygous female mice with a brain-specific deletion of the ChRF ATRX implicated also this ChRF in spatial, contextual fear and novel object recognition (NOR) memory (Tamming et al., 2017).

ChRFs belong to the SNF2 superfamily of ATPases that comprises 23 structurally distinct subfamilies in mammals (Flaus et al., 2006). The most well-studied subfamilies are the SWI/SNF, the ISWI, the INO80 and the CHD subfamilies (Becker and Workman, 2013). Chromodomain-helicase-DNA binding protein 1 (Chd1) is the name-giving member of the CHD subfamily. At the biochemical level, it can assemble the four core histones but not linker histone H1 into regularly spaced nucleosomal arrays (Lusser et al., 2005; Torigoe et al., 2013; Lieleg et al., 2015), and it can slide nucleosomes along the DNA (Stockdale et al., 2006; Rippe et al., 2007; Levendosky et al., 2016; Qiu et al., 2017). Chd1 has two chromodomains that, in the case of the human protein, recognize methylated lysine four at histone H3 (Flanagan et al., 2005; Sims et al., 2005). Several recent structural studies provided detailed mechanistic insights into these properties of Chd1 (Farnung et al., 2017; Nodelman et al., 2017; Sundaramoorthy et al., 2017). On the cellular level, Chd1 was found to have transcription-independent functions, such as in global histone H3.3 incorporation into paternal chromatin in flies (Konev et al., 2007) or in DNA damage repair (Kari et al., 2016; Rüthemann et al., 2017; Shenoy et al., 2017). Importantly, Chd1 also is a critical regulator of transcription initiation and elongation (Marfella and Imbalzano, 2007; Mills, 2017). For example, Chd1 promotes RNAP II promoter escape by remodeling the +1 nucleosome at the transcriptional start site (TSS; Skene et al., 2014). Knock-out of Chd1 in the mouse is lethal due to a requirement of Chd1 for epiblast growth, and endothelia-specific deletion of *Chd1* severely impairs hematopoiesis. Both phenotypes appear to be due to a global reduction in transcriptional output (Guzman-Ayala et al., 2015; Koh et al., 2015). Interestingly, missense mutations in several human patients have recently linked CHD1 to a genetic disorder characterized by autism, speech apraxia, developmental delay and facial dysmorphic features (Pilarowski et al., 2018). The role of CHD1 in the disease mechanism, however, has not been studied.

We have previously generated a mouse line that carries a deletion of exon 2 of the *Chd1* gene (*Chd1*^Δ2/Δ2^). The Chd1 protein expressed in mutant mice (Chd1ΔSRR) lacks 100 amino acids of a serine-rich region in the N-terminus but still contains the chromo-, ATPase and DNA-binding domains and is active in *in vitro* chromatin assembly assays (Piatti et al., 2015). The N-terminal region is subject to extensive phosphorylation (Piatti et al., 2015) and can serve as an interaction module for other proteins (Kelley et al., 1999). Thus, lack of the N-terminus may lead to deficits in Chd1 function. In contrast to *Chd1*^−/−^ mice (Guzman-Ayala et al., 2015), *Chd1*^Δ2/Δ2^ mice are viable and fertile. Embryonic stem cells derived from these mice, however, exhibited aberrant preferential differentiation into neuronal cells under *ex vivo* conditions (Piatti et al., 2015). Our previous study has shown that Chd1 is broadly expressed in the adult mouse brain; during fear extinction learning, it is dysregulated in the ventral hippocampus of the extinction-deficient mouse strain 129S1/SvImJ (Wille et al., 2015) indicating a possible involvement in learning and memory mechanisms.

Because the *Chd1*^Δ2^ mutation causes no obvious developmental defects, we reasoned that the *Chd1*^Δ2/Δ2^ mouse is a suitable model to study a potential involvement of Chd1 in transcription regulatory processes that occur during memory formation and maintenance in the adult brain. Using a series of tests for object recognition and spatial memory, analysis of expression profiles of several critical immediate early genes (IEG) and chromatin immunoprecipitation (ChIP), we discovered that Chd1 is important in particular for spatial memory formation, which correlates with transcriptional dysregulation of the IEGs *early growth response 1* (*Egr1*) and *activity-regulated cytoskeleton-associated* (*Arc*) in *Chd1*-mutant hippocampi suggesting that the N-terminal serine-rich domain of Chd1 is necessary for the transcription-regulatory properties of Chd1 in this context.

## Materials and Methods

### Animals and Husbandry

Young (10 ± 2 weeks) male mice of *C57BL/6N* (obtained from Charles River and Taconic, Germany), *Chd1*^flox/flox^ and *Chd1*^Δ2/Δ2^ (Piatti et al., 2015) strains bred in the animal facility of the Medical University of Innsbruck were used for this study. The animals were housed in groups of 2–3 per cage with a 12-h light/dark cycle (lights on at 7:00 AM) under temperature—(22 ± 2°C) and humidity—(50%–60%) controlled conditions and *ad libitum* access to food and water. *Chd1*^flox/flox^ and *Chd1*^Δ2/Δ2^ strains were bred separately and back-crossed to *C57BL/6N* mice after 5–7 generations. Sibling mating was strictly avoided. Nevertheless, this strategy harbors the theoretical possibility that second site mutations other than the intended might account for the phenotypical differences observed. However, since the first memory tests were performed within four generations of establishing the lines in late 2013, we consider this possibility extremely unlikely. Moreover, the results of the analyses did not change over a time span of almost 4 years (last memory test was performed in June 2017). This study was carried out in accordance with national Austrian law. The protocol was approved by the Austrian Animal Experimentation Ethics Board (Bundesministerium für Wissenschaft Forschung und Wirtschaft, Kommission für Tierversuchsangelegenheiten). Every effort was taken to minimize the number of animals used in the experiments.

### Behavior and Cognition Experiments

#### Object Location and Novel Object Recognition Paradigms

For both paradigms, mice were handled for 1–2 min and then habituated to the experimental apparatus (41 × 41 × 41 cm open field arena containing home-cage floor bedding and illuminated to 150 Lux; Tru Scan, Coulbourn Instruments, Holliston, MA) devoid of objects for 5 min during three consecutive days. Training in the object location memory task (OLM) was conducted by placing the animals into the experimental apparatus containing two identical objects (blue colored Lego Duplo blocks 2.5 × 2.5 × 5 cm) and allowing them to explore for 10 min before returning to the home cage. During the short-term (1 h after training) or long-term (24 h after training) memory retrieval tests, mice were placed in the experimental apparatus for 5 min. For assessment of OLM, one object was placed in the same location as during the training trial, and one object was placed in a new location in the middle of the box (Marschallinger et al., 2015). For the NOR test one familiar object and a new object (100 ml glass beaker) were placed in the same locations as during the training trial (Jaitner et al., 2016). Exploration was scored when the mouse’s nose touched the object. All training and testing trials were videotaped and analyzed by individuals blind to the genotype of the subjects. The time of object exploration was recorded and is expressed as percent exploration time of each object out of total exploration time. A discrimination index was calculated by subtracting the time spent exploring the familiar object (*t*_fam_) from the time spent exploring the novel object or the object in the novel location (*t*_nov_) and dividing by total exploration time [DI = (*t*_nov_ − *t*_fam_)/(*t*_nov_ + *t*_fam_)]. Animals that explored less than 3 s total for both objects during either training or testing were removed from the analysis (Vogel-Ciernia et al., 2013). Two out of 30 *Chd1*^flox/flox^, 2 out of 29 *Chd1*^Δ2/Δ2^ and 0 out of 9 *C57BL/6N* mice were excluded according to this criterium.

#### Barnes Maze

The Barnes Maze (BM) test was carried out with 60 lux illumination on a flat circular table (100 cm diameter; 120 cm height) with 20 circular holes (3.5 cm diameter) that were equally distributed around the perimeter. Only one hole allowed the mouse to exit the maze into a dark escape box, the position of which was kept constant during the entire experiment. Four visual cues were positioned around the maze at an interval of 90°. Mice were transferred into the anteroom of the testing facility 24 h before habituation to the maze. During habituation on day 1, each mouse was placed onto the maze for 5 min to explore the maze with the target hole open. During acquisition trials at days 2–5, the animal was placed into the maze for a maximum of 3 min. If the mouse found the target hole, the escape box was closed and the animal was kept in there for 2 min to let it associate the escape box as a secure place. If the animal did not find the target hole during the allotted time, it was gently guided to the hole. Three trials a day were conducted with 20–30 min inter-trial intervals when mice were returned to the home cage. On day 6 (assessment of short-term memory) and on day 13 (long-term memory), respectively, all holes were closed and the mouse was free to explore the maze for 5 min. Between the two test sessions animals were kept in their home cages. All trials were recorded by a camera and evaluated by an experimenter blinded to the genotype of the animals using Videomot (TSE) software. For evaluation, the number of primary errors (visits to wrong holes before finding the target hole) and the primary latency were determined. For the latter, the board was divided into quadrants, and the time spent in each was measured. The quadrant containing the previously open escape hole is referred to as q1, which is flanked by q2 and q4, while q3 is opposing q1.

#### Visual-Cliff and Light-Dark Test

Overall visual ability was tested in both genotype groups using a visual cliff test (as described in Crawley, 1999). The number of mice stopping or not stopping at the cliff was used as the defining variable for each genotype (*n* = 13/group). Explorative behavior in a brightly lit area (400 lux) was investigated with a light-dark apparatus (Crawley, 1999): a black box was inserted into the open field arena covering one third of the space. Time spent and distance traveled were measured over a 10 min period in the open area. To reach the larger bright compartment defined as open area, the mouse had to leave the dark area completely and one small field at the entrance of the black box was defined as transition zone. The entire session was recorded with a video camera on top of the apparatus, and the times spent in each zone were analyzed using a tracking program (Videomot, TSE).

#### Spontaneous Alternation Test (Y-Maze)

The test was conducted using a symmetrical Y-maze. The dimensions of each arm were 35 × 5 × 10 cm, and the walls of each arm were decorated with different black and white patterns. Illumination in the testing area was 50 lux. The test was carried out in a single trial of 8 min, in which the mouse was allowed to explore all three arms. An alternation was defined as a triplet of sequential entries into a different arm (ABC). The alternation score was calculated by dividing the number of correct triplets by the total number of alternations.

### Histology

*Chd1*^Δ2/Δ2^ (*n* = 5) and *Chd1*^flox/flox^ (*n* = 5) mice were sacrificed and brains were fixed by transcardial perfusion with 4% paraformaldehyde in phosphate buffered saline (PBS pH 7.2). The brains were kept in Tris-buffered saline (TBS) + 0.1% sodium azide until embedding into 10% gelatin. Slices of the entire dorsal hippocampus (40 μm) were cut using a vibratome, mounted on gelatin coated slides and subjected to Nissl staining. Pictures were taken at 1.25× and 5× magnification and overall hippocampal anatomy was evaluated by an experimenter blinded to the genotype of the animals.

### Tissue Sampling

*Chd1*^Δ2/Δ2^ and *Chd1*^flox/flox^ mice were sacrificed 30 min after exposure to the OLM test arena, OLM training session or the long-term OLM test trial, respectively, and brains were removed. Dorsal hippocampi (dHC) of both hemispheres were dissected, snap frozen and stored at −80°C. Whole hippocampi were dissected for Western blot analyses.

### Reverse Transcription Real-Time PCR (RT-qPCR)

Quantitative RT-PCR was performed as described before (Wille et al., 2015). Briefly, frozen hippocampus samples were pulverized using the Cryoprep system (Covaris), and total RNA was extracted using Tri Reagent (Sigma Aldrich) according to manufacturer’s instructions followed by DNase I digestion and spin column clean-up (Jena Analytik). Up to 5 μg of RNA were reverse-transcribed using the GoScript Reverse Transcription System (Promega). Real time PCR was performed in triplicate using Luna^®^ Universal qPCR Master Mix (New England Biolabs) with 25 ng cDNA and 0.4 μM of target-specific primers in a StepONE Plus instrument (Applied Biosystems). Primer sequences are shown in Table 1. ΔCt values (using TBP transcripts as the reference) were calculated and results are presented as 2^−ΔΔCt^ ± SEM.

**Table 1:** Sequences of primers used in reverse transcription quantitative PCR (RT-qPCR) and ChIP-qPCR.

RT-qPCR	Forward primer	Reverse primer
*Bdnf*	5′CGGACCCATGGGACTCTGGA	5′GTTGGGCCGAACCTTCTGGT
*Arc*	5′CGCAGAAGCAGGGTGAACCA	5′TCCTCCTCCTCAGCGTCCAC
*Egr1*	5′GCGCCCACCTTTCCTACTCC	5′CCAGGCTCAGGTCTCCCTGT
*cFos*	5′AGCAACGTGGAGCTGAAGGC	5′TGTGCAGAGGCTCCCAGTCT
*Grin2A*	5′AACTACAAGGCCGGGAGGGA	5′ACTGGAGCAGAGCGAGGTCA
*TBP*	5′TAATCCCAAGCGATTTGCTGC	5′TTCACTCTTGGCTCCTGTGC
**ChIP qPCR**	**Forward primer**	**Reverse primer**
amplicon centered at position		
−180 (Egr1)	5′GCTTTCCAGGAGCCTGAGC	5′AGCCCTCCCATCCAAGAGT
−126 (Egr1)	5′GTGCCCACCACTCTTGGAT	5′GCAGGAAGCCCTAATATGGA
−69 (Egr1)	5′GCCGGTCCTTCCATATTAGG	5′TCAAGGGTCTGGAACAGCAC
38 (Egr1)	5′CGAGAGATCCCAGCGCGCAG	5′TCTTGCGGCGGCGGAAGCTG
171 (Egr1)	5′GGGGCCCACCTACACTCC	5′GTTGGCCGGGTTACATGC
2204 (Egr1)	5′CAGAAGCCCTTCCAGTGTCG	5′GATGGGTAGGAGGTAGCCAC
2918 (Egr1)	5′CCCTTCAGCGCTAGACCATC	5′GCTGTACAAAGATGCAGGGC
*β-actin*	5′ACGCCATCCTGCGTCTGGAC	5′ATGACCTGGCCGTCAGGC

### ChIP Assays

For ChIP assays using the mouse progenitor hepatocyte cell line MLP29, cells were cultured and treated with 50 nM 12-O-tetradecanoylphorbol-13-acetate (TPA) for 30 min as described previously (Tur et al., 2010). Cells were crosslinked with 1% formaldehyde in PBS for 6 min at room temperature with constant shaking, and the crosslinking reaction was stopped with 0.125 M glycine in PBS. Cells were collected, washed with PBS and suspended in cell lysis buffer (5 mM HEPES, 85 mM KCl, 0.5% NP40, pH 8.0) containing 2 μl/ml protease inhibitor cocktail (Sigma). Samples were then incubated on ice for 15 min, and nuclei were recovered by centrifugation at 3500×*g* for 5 min at 4°C. The sediment was gently resuspended in nuclear lysis buffer (50 mM Tris-HCl, 10 mM EDTA, 1% SDS pH 8.1). For fragmentation chromatin was subjected to 3 cycles of 5 min sonication (30 s on, 30 s off) in a Bioruptor Plus instrument (Diagenode). Under these conditions, the average size of chromatin fragments was 300 ± 200 bp. ChIP analyses as well as the qPCR amplification of the DNA recovered from the immunoprecipitates were carried out essentially as described by Riffo-Campos et al. (2015), with the primers shown in Table 1.

For ChIP from hippocampus, *C57BL/6N* male mice were either taken directly from the home cage (*n* = 4) or subjected to the training session for the OLM test (*n* = 4). Twenty minutes after completion of the training session, the animals were sacrificed and hippocampi were dissected and flash frozen. Hippocampi from each mouse were processed separately for crosslinking and chromatin preparation using the Zymo-Spin ChIP Kit (Zymo Research) exactly following the manufacturer’s ChIP protocol for mouse tissue. Before the IP, sheared chromatin from the four mice of each group was pooled to obtain enough material, the samples were precleared for 60 min at 4°C with ZymoMag Protein A magnetic beads (Zymo Research), followed by incubation of 15 μg chromatin with 5 μl α-CHD1 antibody or no antibody (NA) over night at 4°C. Incubation of antibody and NA IP samples with magnetic beads, stringency washes and DNA purification was performed according to the Zymo-Spin ChIP Kit protocol. The antibody used for ChIP assays was α-CHD1 (D8C2-4351, Cell Signaling Technology). To correct for potentially different amounts of input chromatin, we measured β-actin and adjusted the signals in the IP fractions using the antibody or the non-antibody (NA) control accordingly. IP signals were corrected for background (NA) and normalized to the input.

### Protein Extract Preparation and Immunoblotting

Preparation of nuclear protein extracts and immunoblotting were performed exactly as described in Wille et al. (2015). Chd1 was detected using a commercially available antibody at 1:1,000 dilution (Proteintech 20576-1AP). Recombinant purified full-length Chd1 and Chd1ΔSRR proteins (Piatti et al., 2015) were used as size references on Western blots.

### Statistics

#### NOR and OLM Tests

Initially all data were tested for normal distribution (using Shapiro-Wilk test) followed by testing for homoscedasticity. As parametric distributions were revealed for all data, exploration time data (expressed as %) was further subjected to an outlier analysis using Grubb’s test resulting in the exclusion of one *Chd1^flox/flox^* animal from the short-term NOR group from further analysis. All other data were statistically analyzed using two-way ANOVA with genotype and object/position as independent variables and exploration time as dependent variable. A Duncan’s *post hoc* comparisons test was applied whenever possible (Sigmaplot 12.0, UK). For the discrimination index, an unpaired two-tailed student’s *t*-test was used. All data are presented as mean ± SEM. Statistical significance was set at *p* < 0.05.

#### Visual Cliff, Light-Dark Test, Barnes Maze Test, Y-Maze Test

Data obtained from visual cliff and Y-maze tests were analyzed using Chi-squared test and Mann-Whitney *t*-test, respectively, comparing the two genotype groups. The total times spent in light or dark compartments of the light-dark test were compared using 2-way ANOVA (genotype and preferred area). The learning curves during the BM were analyzed with 2-way ANOVA for repeated measures (genotype and trials). The time spent in each quadrant during the short-term and long-term memory tests (BM) were analyzed for the two genotypes separately using a 1-way ANOVA and Dunnett’s multiple comparisons. All data are given as mean ± SEM and were analyzed using GraphPad Prism 7 with a significance threshold of *p* < 0.05.

#### RT-qPCR

Mean 2^−ΔΔCt^ ± SEM values of the indicated genes were calculated for the different test groups (*n* values are given in the caption of Figure 6) and analyzed by multiple pairwise *t*-test with Holm-Sidak correction for multiple testing using GraphPad Prism 7. Correlations between discrimination index and gene expression were calculated using the built-in correlation function of Prism 7. Significance threshold was *p* < 0.05 for all calculations.

**Figure 1:**
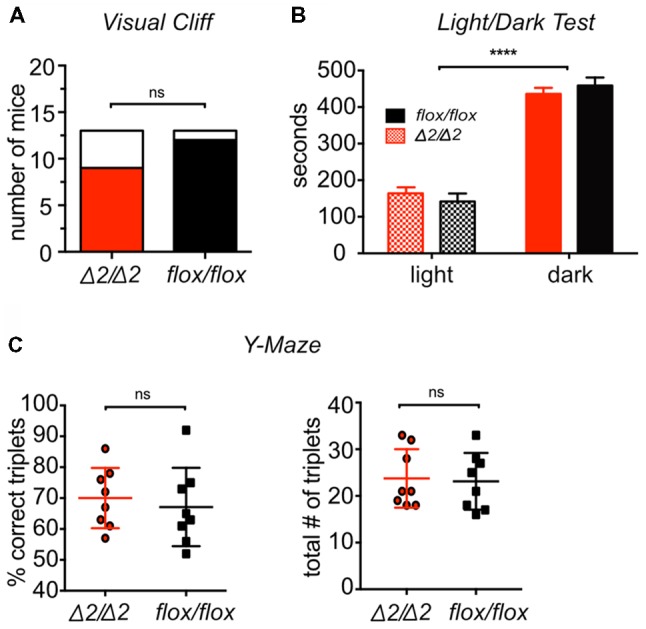
*Chd1*-mutant mice have normal vision, anxiety, working memory and motivation to explore the environment. **(A)** Visual ability was tested by the visual cliff test. The number of mice stopping (colored) or not stopping (white) at the cliff is shown for each genotype. No statistically significant difference was detected (Chi-squared test, *p* > 0.05; *n* = 13/group). **(B)** Results of light/dark test for visual ability and anxiety are expressed as time spent in either the light or the dark compartment of the test set-up. Both groups preferred the dark compartment without differences between them (two-way ANOVA; compartment effect: *F*_(1,48)_ = 231.8, *p* < 0.0001; genotype effect: *F*_(1,48)_ = 0, *p* > 0.05; *n* = 13/group). **(C)** Working memory and motivation to explore were tested by a spontaneous alternation test using a Y-maze. An alternation was defined as a triplet of sequential location visits. Results are expressed as the fraction of “correct” triplets (sequential visits to three unique locations; left graph) out of all alternations and total number of triplets (right graph). No significant difference was detected for the two genotypes (Mann-Whitney test, *p* > 0.05 for left graph; *p* > 0.05 for right graph, *n* = 8/group). Results were considered significant at *p* < 0.05 (****p < 0.0001; ns, non-significant). Means ± SEM are shown.

**Figure 2:**
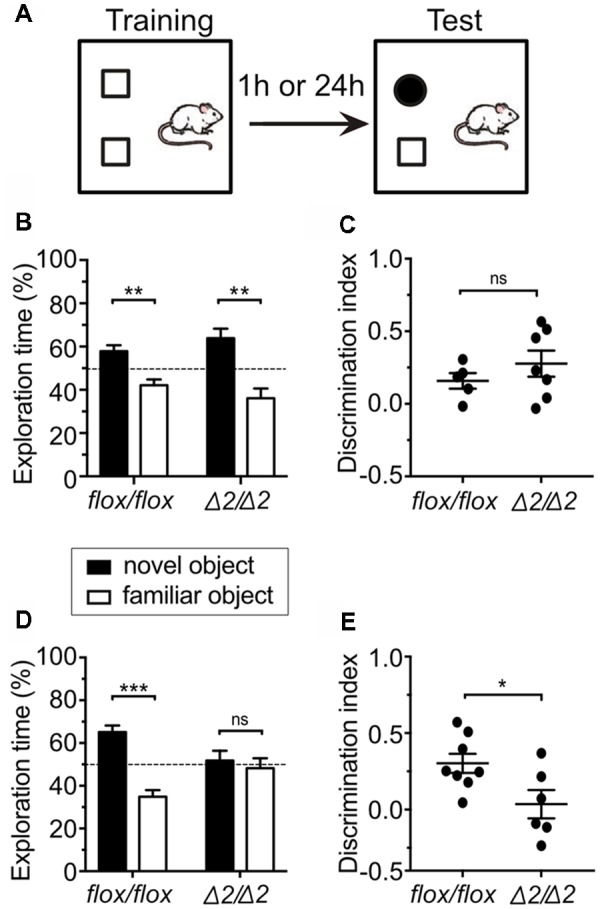
Dysfunctional long-term object recognition memory in *Chd1*^Δ2/Δ2^ mice. **(A)** Experimental setup for the Novel Object Recognition (NOR) test. Mice were tested either for short-term memory (1 h) or long-term memory (24 h). **(B)** Exploration time of both objects during the test session for short-term memory was measured and is expressed in % of total exploration time. The dashed line indicates the chance level (50%). Two-way ANOVA revealed a significant object effect (*F*_(1,20)_ = 27.993, *p* < 0.001) for both *Chd1*^flox/flox^ and *Chd1*^Δ2/Δ2^ mice. Duncan’s *post hoc* test revealed that both *Chd1*^flox/flox^ (*p* < 0.05) and *Chd1*^Δ2/Δ2^ (*p* < 0.001) preferred the novel object instead of the familiar object. **(C)** The discrimination index for the novel object was calculated (see “Materials and Methods” section) and plotted for each animal. Statistical significance was calculated by unpaired *t*-test. **(D)** In the long-term memory test (24 h), a significant genotype × object interaction effect was observed (*F*_(1,24)_ = 12.355, *p* < 0.01). *Post hoc* analysis revealed that while *Chd1*^flox/flox^ preferred the novel object instead of the familiar object (*p* < 0.001), *Chd1*^Δ2/Δ2^ displayed similar preference for both objects (*p* > 0.05). **(E)** Same as in **(C)** for long-term memory (24 h) testing. Significance threshold was set to *p* < 0.05 (*p < 0.05, **p < 0.01, ***p < 0.001, ns, non-significant). For **(B,C)** *n* = 5 (*Chd1*^flox/flox^) and 7 (*Chd1*^Δ2/Δ2^), respectively. For **(D,E)** *n* = 8 (*Chd1*^flox/flox^) and 6 (*Chd1*^Δ2/Δ2^), respectively. Means ± SEM are shown.

**Figure 3:**
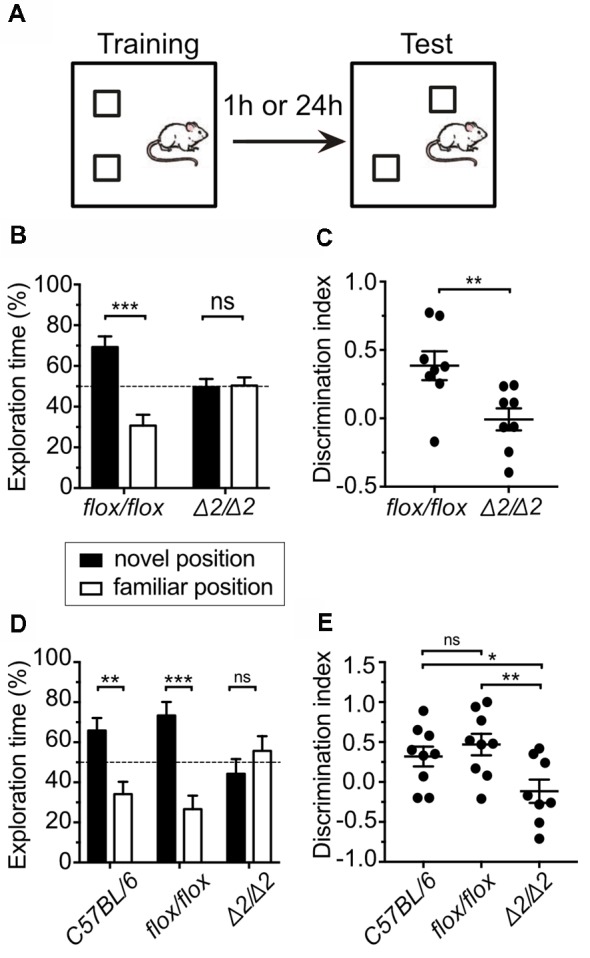
*Chd1*^Δ2/Δ2^ mice show deficits in short and long-term spatial memory. **(A)** Experimental setup for the Object Location Memory (OLM) test. Mice were tested either for short-term (1 h) or long-term spatial memory (24 h). **(B)** Exploration time of both objects during the test session for short-term memory was measured and is expressed in % of total exploration time. The dashed line indicates the chance level (50%). Two-way ANOVA revealed a significant genotype × position interaction effect (*F*_(1,28)_ = 17.578, *p* < 0.001). Duncan’s *post hoc* test revealed that while *Chd1*^flox/flox^ mice preferred the object at the novel position instead of the familiar position (*p* < 0.001), *Chd1*^Δ2/Δ2^ animals displayed similar preference for both the positions (*p* > 0.05). **(C)** The discrimination index for the object in the novel location was calculated (see “Materials and Methods” section) and plotted for each animal. Statistical significance was calculated by unpaired *t*-test. **(D)** In the long-term memory test (24 h), a significant genotype × position interaction effect (*F*_(2,46)_ = 9.7144, *p* < 0.001) was observed. *Post hoc* test revealed that while both *C57BL/6N* and *Chd1*^flox/flox^ preferred the object at the novel position instead of the familiar position (*p* < 0.01 and *p* < 0.001 respectively), *Chd1*^Δ2/Δ2^ displayed similar preference for both positions (*p* > 0.05). *C57BL/6N* mice were used as an additional control group. **(E)** Same as in **(C)** for long-term memory (24 h) testing. *C57BL/6N* mice were used as an additional control group. Significance threshold was set to *p* < 0.05 (*p < 0.05, **p < 0.01, ***p < 0.001, ns, non-significant). For **(B,C)** *n* = 8 for both groups. For **(D,E)** *n* = 9 (*C57BL/6N*), *n* = 9 (*Chd1*^flox/flox^) and 8 (*Chd1*^Δ2/Δ2^), respectively. Means ± SEM are shown.

**Figure 4:**
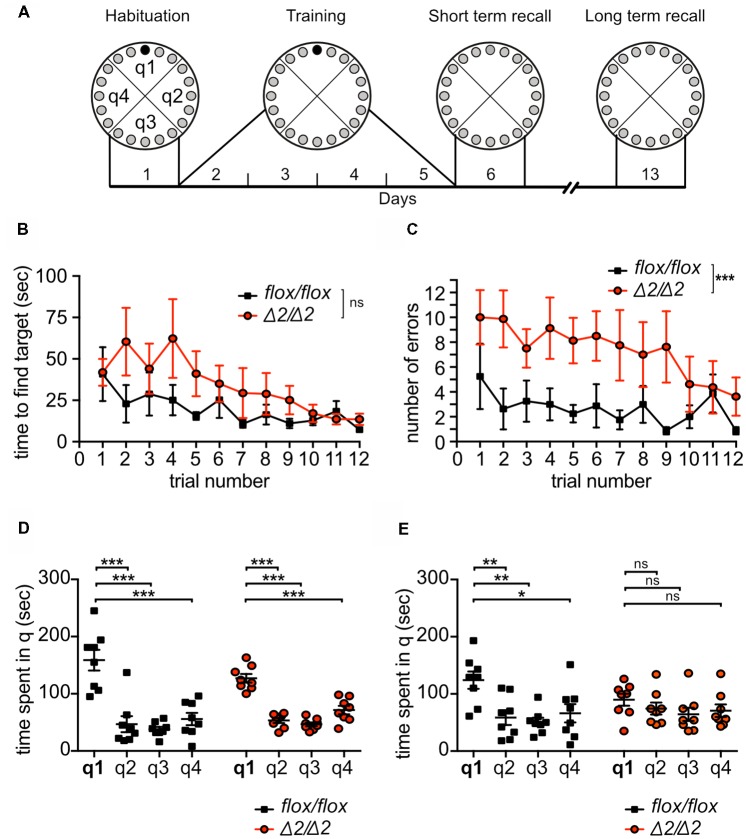
Learning and long-term memory deficits of *Chd1*^Δ2/Δ2^ mice in the Barnes Maze (BM) test. **(A)** Schematic representation of BM experimental set-up. Animals were trained during 4 days with three training trials on each day to find the target hole (black circle). On day 6, all holes were closed and animals were tested for short-term memory. On day 13, animals were tested for long-term memory. **(B,C)** Learning was monitored by recording the time required to find the target hole (latency; **B**) and by determining the number of errors before finding the target hole **(C)** during each training trial. Statistical analysis was performed by two-way repeated measures ANOVA (*n* = 8/group). **(D,E)** Short term memory **(D)** and long-term memory **(E)** were assessed by recording the time spent in each quadrant in the test trial at day 6 **(D)** and 13 **(E)**. Statistical analysis in **(D,E)** was performed by one-way ANOVA for each genotype (*n* = 8 per group). q1–4, quadrants 1–4; q1 is the target quadrant. Means ± SEM are shown. Statistical significance threshold was set to *p* < 0.05 (*p < 0.05, **p < 0.01, ***p < 0.001, ns, non-significant).

**Figure 5:**
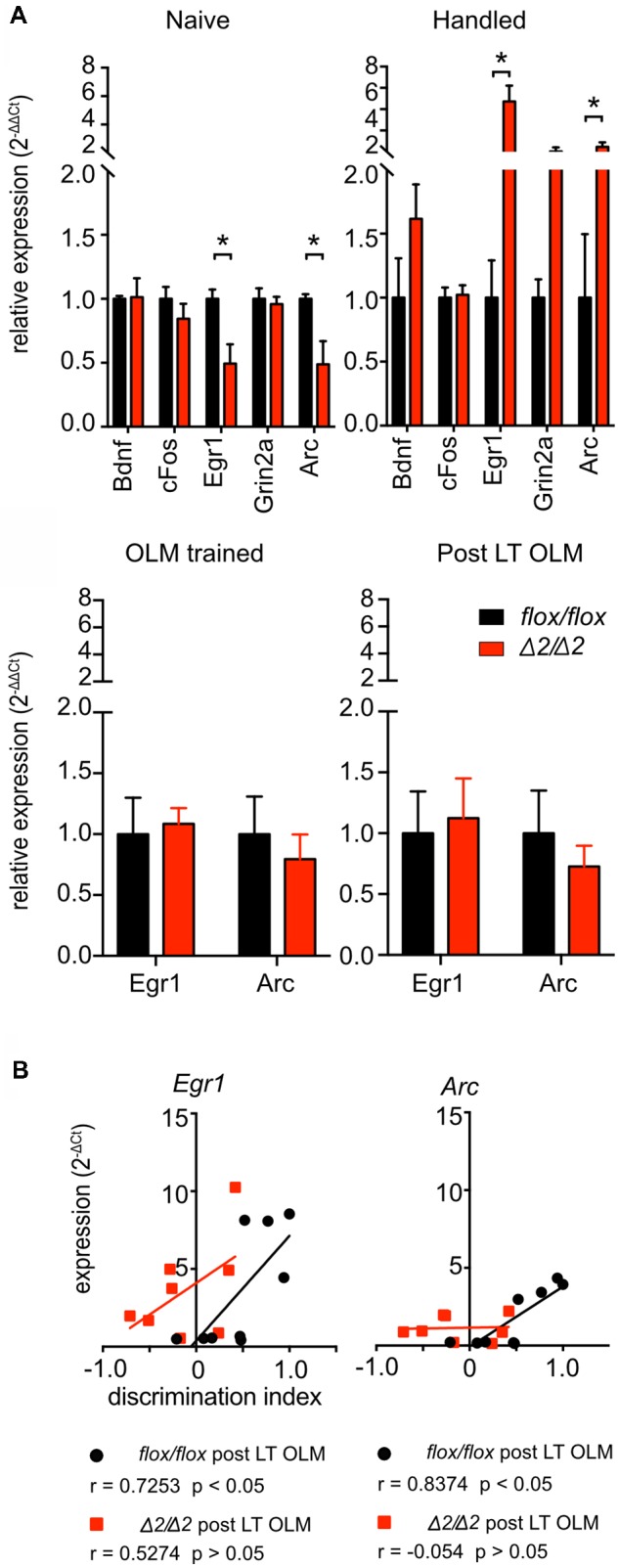
Dysregulation of immediate early genes (IEG) in the hippocampus of *Chd1*^Δ2/Δ2^ animals. **(A)** Reverse transcription real-time PCR was performed on RNA from dorsal hippocampi (dHC) isolated from *Chd1*^Δ2/Δ2^ and *Chd1*^flox/flox^ mice kept in their home cage (“naïve”; *n* = 4/4), mice from both groups that were habituated to the test arena but not trained or tested (“handled”; *n* = 3–4/group), animals that were sacrificed 30 min after long-term OLM training (“OLM trained”; *n* = 4/4) or 30 min after long-term OLM testing (“Post LT OLM”; *Chd1*^flox/flox^ *n* = 9, *Chd1*^Δ2/Δ2^ *n* = 8). **(B)** Expression of *Egr1* and *Arc* correlates with the performance (discrimination index) of the respective *Chd1*^flox/flox^ mice in the OLM test, while no such correlation was detected for *Chd1*^Δ2/Δ2^ animals. Correlation coefficients (*r*) and *p* values are shown in the figure legends. Statistical analysis was performed by pairwise *t*-test with Holm-Sidak correction for multiple testing for **(A)** and using the built-in correlation analysis tool of GraphPad Prism 7 for **(B)**. *p* < 0.05 was considered significant (*p < 0.05).

**Figure 6:**
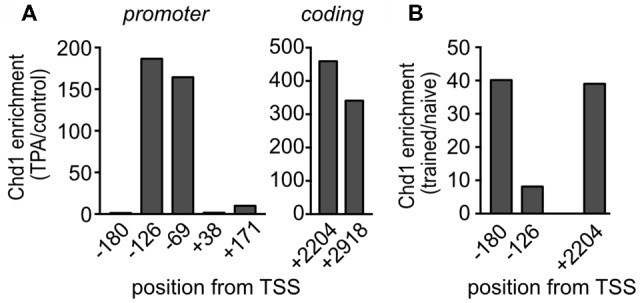
Chd1 accumulates at the *Egr1* gene locus upon stimulation. **(A)** *Egr1* expression was induced with 12-O-tetradecanoylphorbol-13-acetate (TPA) in MLP29 cells, and chromatin immunoprecipitation (ChIP) analysis was performed with an antibody against Chd1 before and at 30 min after induction. **(B)** Hippocampi were isolated from naïve *C57BL/6N* mice and from mice 20 min after completion of the training session in the OLM paradigm, and ChIP analysis was performed on chromatin pools from HC of four mice per group. Antibody-bound DNA was detected by qPCR with primers spanning the indicated positions at the *Egr1* genomic locus (numbering relative to the transcriptional start site, TSS). Enrichment of signals from TPA induced vs. non-induced conditions **(A)** and naïve vs. trained animals **(B)**, respectively, is shown for amplicons located in the promoter and coding region, respectively.

## Results

### Mutation of *Chd1* Does Not Affect Vision, Motivation to Explore, Anxiety or Working Memory

Although *Chd1*^Δ2/Δ2^ mice do not have any obvious phenotypes and appear healthy, we first examined brain (in particular hippocampus) morphology by Nissl staining of mutant mouse brain sections. As expected, morphology appeared normal (Supplementary Figure S1) suggesting that any results obtained from the following experiments were likely not affected by neurodevelopmental aspects. To study a potential involvement of Chd1 in learning and memory processes, we used several established cognition and behavior tests, including the NOR test, the OLM test and the BM paradigm with *Chd1*^Δ2/Δ2^ and the corresponding *Chd1*^flox/flox^ control mice. To ensure validity of the results obtained from the cognition tests, we first examined potential confounding phenotypes, such as poor visual abilities, reduced motivation to explore an environment, impaired working memory or elevated anxiety. To this end, the mice were subjected to a visual cliff test, light/dark test and a spontaneous alternation test based on the Y-maze. The performance of *Chd1*^Δ2/Δ2^ mice in visual cliff and light/dark tests was indistinguishable from *Chd1*^flox/flox^ control animals (Figures 1A,B) indicating that vision is not impaired by the N-terminal truncation of Chd1. The light/dark test also measures trait anxiety of animals, as anxious mice tend to spend increased amounts of time in the dark area of the arena (Crawley and Goodwin, 1980). Thus, the results suggest that *Chd1*^Δ2/Δ2^ mice do not suffer from elevated anxiety. Finally, in the Y-maze, the mice were allowed to explore the arms of a three-armed symmetrical maze for 8 min. Normal working memory is reflected by a high frequency of subsequent entries into each different arm (ABC; “correct triplet”), while motivation to explore is measured in the total number of entries into any arm. Both *Chd1*^Δ2/Δ2^ and *Chd1*^flox/flox^ mice behaved similarly with respect to these criteria suggesting functional working memory and motivation to explore for *Chd1*^Δ2/Δ2^ mutant animals (Figure 1C).

### N-Terminally Truncated Chd1 Causes Defects in Long-Term Memory

To study the learning and short-term memory abilities of *Chd1*^Δ2/Δ2^ mice, we used the NOR test, in which the mouse is placed into an arena containing two different objects and allowed to explore these objects for 10 min, before being transferred back to the home cage. After a delay of 1 h, the animal was returned to the arena in which one of the objects had been exchanged for a new one to assess short-term memory (Figure 2A). Functional short-term memory is reflected by the fact that the animal spends more time exploring the new object than it does with the familiar one. *Chd1*^Δ2/Δ2^ and *Chd1*^flox/flox^ mice showed significant preference for the new object over the familiar one, and exploration time was higher than the chance value of 50% (Figure 2B). There was no significant difference with respect to the genotype indicating that learning and short-term memory abilities were normal in *Chd1*-mutant mice (Figures 2B,C). The results further imply that there is no difference in exploratory drive between the two strains (see Table 2 for total exploration times). By contrast, when the mice were returned to the arena 24 h after the training, only control but not *Chd1*^Δ2/Δ2^ mice displayed novel object preference (Figure 2D, Table 2). This difference was also evident when comparing discrimination indices of *Chd1*-mutant and control mice (Welch’s test, *p* < 0.05; Figure 2E). Hence, these results point toward a deficit in long-term memory in *Chd1*^Δ2/Δ2^ mice.

**Table 2:** Total exploration times (seconds) of animals subjected to novel object recognition (NOR) and object location memory (OLM) tests.

	*Chd1*^flox/flox^	*Chd1*^Δ2/Δ2^	*C57BL/6N*
NOR short term	10.4 ± 2.45	8.38 ± 1.94	n.a.
NOR long term	9.22 ± 1.62	9.23 ± 4.28	n.a.
OLM short term	8.56 ± 1.89	5.23 ± 0.44	n.a.
OLM long term	4.75 ± 0.4	5.54 ± 0.67	5.46 ± 0.98

*n.a., not available*.

### *Chd1* Mutation Severely Impairs Short- and Long-Term Spatial Memory

Next, we investigated if spatial memory is affected in *Chd1*^Δ2/Δ2^ animals. To this end, we subjected the mice to an OLM task. The training phase of this paradigm uses the same set-up as the NOR test, but upon testing, the second object is not replaced but moved to a different location within the arena (Figure 3A). Mice with intact spatial memory will spend more time exploring the object in the new location compared to the object in the familiar location. Interestingly, *Chd1*-mutant animals were unable to remember the location of the object in the short-term OLM test as they spent equal time with both objects when placed back into the arena 1 h after the training trial, while *Chd1*^flox/flox^ control mice showed clear preference for the newly positioned object (Figures 3B,C, Table 2). We obtained similar results when we tested for long-term spatial memory by returning the mice into the test arena 24 h after training illustrated by the exploration time (Figure 3D, Table 2) and discrimination index data (Figure 3E) of control and mutant animals. In this experiment, a group of *C57BL/6N* mice was included as an additional wild-type control confirming that the *Chd1*^flox/flox^ strain behaves like wild type (Figures 3D,E, Table 2). These results indicate that Chd1 is involved in the formation of short and long-term spatial memory and that the N-terminal serine-rich region is necessary for this function.

To further corroborate a role for Chd1 in spatial memory formation and/or consolidation, we examined spatial reference learning and memory using the BM (Sunyer et al., 2007), which in contrast to the OLM involves an extended acquisition period (Figure 4A). During the 4 days of training, primary latency to find the escape hole and the number of errors before finding the escape hole were recorded at each trial (3 trials per day). Monitoring primary latency during the training period showed progressive and significant decrease in latency across trials for both *Chd1*^Δ2/Δ2^ and *Chd1*^flox/flox^ mice (two-way ANOVA repeated measures: training effect: *F*_(11,154)_ = 2.807, *p* < 0.01). Although *Chd1*^Δ2/Δ2^ animals appeared to require more time to find the target hole during the first 2 days of training than *Chd1*^flox/flox^ mice, they reached similar performance during later trials, overall resulting in no statistically significant differences between the strains (two-way ANOVA repeated measures: genotype effect: *F*_(1,14)_ = 3.1, *p* > 0.05; time effect: *F*_(11,154)_ = 2.807, *p* = 0.0023, interaction: *F*_(11,154)_ = 0.909, *p* = 0.534; Figure 4B). By contrast, *Chd1*^Δ2/Δ2^ mice made significantly more errors before finding the target hole than *Chd1*^flox/flox^ mice across most of the training trials (two-way ANOVA repeated measures: genotype effect: *F*_(1,14)_ = 18.83, *p* = 0.001; time effect: *F*_(11,154)_ = 1.249, *p* = 0.2597; interaction: *F*_(11,154)_ = 0.6108, *p* = 0.8177; Figure 4C) suggesting a mild impairment of acquisition of spatial memory in *Chd1*^Δ2/Δ2^ mice. On day 6, short-term spatial memory was tested by measuring the time the animals spend in the target quadrant (escape hole closed). Both *Chd1*-mutant and wild-type mice exhibited clear preference for the target quadrant q1 compared to the other quadrants (one-way ANOVA: *F*_(3,28)_ = 19.26, *p* < 0.0001) indicating successful retrieval of short term spatial memory (Figure 4D). When we evaluated long-term memory at day 13 by returning the animals to the BM under the same conditions as in the short-term memory test, *Chd1*^flox/flox^ control animals again spent significantly more time in q1 than in the other quadrants (one-way ANOVA: *F*_(3,28)_ = 6.28, *p* < 0.01; Figure 4E) demonstrating intact spatial long-term memory. In sharp contrast, *Chd1*^Δ2/Δ2^ mice exhibited complete loss of recall as they no longer preferred q1 to the other quadrants (one-way ANOVA: *F*_(3,28)_ = 0.99, *p* > 0.05; Figure 4E).

Taken together, the results from evaluation of object recognition and spatial memory indicate that Chd1 is involved in the formation/recall of memory. Both NOR and OLM tests show that long-term memory is impaired. Moreover, spatial memory appears to be particularly sensitive to *Chd1* mutation, because also short-term memory was affected in the OLM test. Upon increasing stimulation of (spatial) memory formation, such as in the BM when training proceeds over several days, short term recall is possible, while long-term memory is still deficient.

### Dysregulation of IEGs in the Hippocampus of *Chd1*-Mutant Mice

As a ChRF, Chd1 is a central component of the transcription regulatory machinery. Given that we observed impaired memory, in particular, spatial memory abilities of *Chd1*-mutant mice, we sought to determine if the activity of genes linked to learning and memory is disturbed in the dHC, which is the central region for spatial memory processing (Fanselow and Dong, 2010). First, we confirmed by Western blot that wild-type Chd1 is present in the hippocampus of floxed animals (Supplementary Figure S2A). Chd1 protein was also detected in a quantitative mass spectrometry analysis of hippocampal proteins in *C57/BL6N* mice as a protein with average abundance (Supplementary Figure S2C). Likewise, we detected the Chd1ΔSRR mutant protein in hippocampal extracts of *Chd1*^Δ2/Δ2^ mice (Supplementary Figure S2B).

We then analyzed the expression of the IEGs *cFos*, *Egr1* and *Arc* as well as of TrkB receptor ligand *brain derived neurotrophic factor (Bdnf)* and *Grin2a* (encoding N-methyl-D-aspartate receptor subunit 2a) by reverse transcription quantitative PCR (RT-qPCR) analysis. Expression analysis of these genes revealed that in HC of naïve mice, i.e., mice taken from the home cage, the transcript levels of *Egr1* and its direct target gene *Arc* were significantly lower in *Chd1*^Δ2/Δ2^ compared to *Chd1*^flox/flox^ animals, while *Bdnf*, *cFos* and *Grin2a* expression was similar (Figure 5A). When animals from both groups were habituated to a new environment, i.e., the OLM test arena (Figure 5A “handled”), again *Egr1* and *Arc* transcript levels were the only ones that were significantly affected in the dHC, in this case they were increased in mutant vs. control animals (Figure 5A). These results point towards a defect in the regulation of *Egr1* and *Arc1* in the absence of fully functional Chd1. We then assessed *Egr1* and *Arc* expression in mice that underwent training for OLM as described in Figure 3 to determine whether the observed long-term memory deficit of *Chd1*^Δ2/Δ2^ animals involved a potential dysregulation of the IEGs at the stage of memory formation. However, expression levels of *Egr1* and *Arc* did not differ between *Chd1*^Δ2/Δ2^ and *Chd1*^flox/flox^ animals (Figure 5A “OLM trained”). This suggests that if failure to form an OLM in *Chd1*^Δ2/Δ2^ animals is the cause for the observed long-term memory defect, this process is not affected by altered regulation of *Egr1* and *Arc*, because their expression at the training stage is essentially the same in mutant and control animals. Somewhat unexpectedly, we also did not detect expression differences of IEGs in dHC when both groups were analyzed after completion of the long-term OLM paradigm (i.e., at the stage of memory retrieval, Figure 5A “post LT OLM”) although the *Chd1*^Δ2/Δ2^ animals clearly displayed loss of long-term memory in the OLM test (Figures 3D,E). Wild-type mice have been shown to exhibit induction of learning and memory-associated IEGs, such as *Egr1* and *Arc*, upon undergoing memory retrieval in different memory tasks (Minatohara et al., 2015). We therefore examined if there was a correlation between *Egr1* and *Arc* expression levels and the performance of the respective mice in the long-term OLM task in the *Chd1*^flox/flox^ and *Chd1*^Δ2/Δ2^ animals. We found a clear and significant positive correlation of *Egr1* and *Arc* expression with the discrimination index in the OLM task of the respective *Chd1*^flox/flox^ control mice (Figure 5B). By contrast, no correlation between IEG expression and cognitive performance was observed among *Chd1*^Δ2/Δ2^ animals (Figure 5B).

Taken together, the data point towards a role for Chd1 in the regulation of *Egr1* and *Arc* in the HC. This role is not restricted to a particular stage of stimulation, such as exposure to a new environment or memory retrieval, but is already evident in naïve unstimulated animals.

### Chd1 Binds to the *Egr1* Promoter and Coding Region

To determine whether Chd1 regulates *Egr1* in a direct manner, we analyzed if Chd1 is physically present at the *Egr1* gene. To this end, we used the MLP29 cell line, in which the *Egr1* promoter had been extensively characterized with respect to chromatin changes and factor binding in response to transcriptional induction (Tur et al., 2010; Riffo-Campos et al., 2015) to perform ChIP assays for Chd1. These experiments revealed that in the absence of transcription induction, no or very low levels of Chd1 were detectable at different positions within the *Egr1* promoter region (Supplementary Figure S3). By contrast, we observed clear enrichment of Chd1 upon induction of transcription by TPA (Figure 6A, Supplementary Figure S3). Moreover, consistent with a role for Chd1 in transcription elongation (Simic et al., 2003; Lee et al., 2017), TPA also induced accumulation of Chd1 in the gene body of the *Egr1* gene (Figure 6A). Because it is possible that the molecular machinery regulating *Egr1* expression differs between MLP29 cells and the mouse brain, we also performed ChIP assays with HC tissue isolated from naïve wild-type mice and animals that were subjected to the OLM training paradigm described in Figure 3 to induce *Egr1* expression. Due to the limited amount of material available from the tissue samples, we only assessed Chd1 presence at three out of the seven locations shown in Figure 6A. Our data demonstrate that training indeed induces accumulation of Chd1 at the promoter and coding region of *Egr1* in hippocampal tissue (Figure 6B). From these results, we conclude that *Egr1* is a direct target for regulation by Chd1 in cultured cells as well as in the hippocampus. Because *Egr1* and *Arc* are tightly associated with neuronal activity, their dysregulation may underlie the learning and memory defects observed in *Chd1*-mutant mice.

## Discussion

One of the salient features of establishment and consolidation of memory is a change in gene expression programs in various regions of the brain. Synaptic input results in the activation of cellular signaling cascades leading to transcriptional activation of IEGs that in turn orchestrate molecular changes required for synaptic plasticity and neuronal activity (Minatohara et al., 2015). Transcriptional activation (or repression) requires the remodeling of chromatin structure at the promoters and, to some extent, in the coding regions of target genes. Posttranslational modification of histones is intimately linked to activation or repression of transcription. While posttranscriptional modification operates *via* affecting electrostatic interactions between neighboring nucleosomes (e.g., acetylation) or by creating binding sites for the recruitment of transcriptional co-activators/co-repressors (e.g., methylation), transcriptional activation generally requires yet more profound chromatin structure changes, such as repositioning and/or eviction of nucleosomes around the TSS. The latter is elicited by ChRFs that hydrolyze ATP to slide nucleosomes to new positions or to remove them from the TSS allowing access of the RNA polymerase holoenzyme to the promoter and subsequent promoter escape of elongating polymerase, respectively (Venkatesh and Workman, 2015).

In this work, we studied whether mutation of the ATP-dependent remodeling and assembly factor Chd1 interfered with transcription in the brain to an extent that would negatively affect cognitive functions. Our results show that Chd1 is indeed involved in the molecular machinery regulating memory formation. In three different forms of learning, the single trial NOR and OLM tasks as well as in the BM involving extended training, *Chd1*^Δ2/Δ2^ mice exhibited severely impaired long-term memory. By contrast, short term memory was only impaired for object location but not for NOR, spatial navigation (BM) or spontaneous alternation (Y-maze). These results suggest that Chd1 is required for memory retrieval and/or for consolidation of memory during or after the learning process. The fact that in the BM task *Chd1*^Δ2/Δ2^ animals required more training trials to reach the same performance as *Chd1*^flox/flox^ mice (Figure 4C) support a role for Chd1 in memory consolidation. A similar phenotype with respect to short- and long-term memory abilities has been reported for mice lacking the IEG *Egr1* (Jones et al., 2001). Similar to Egr1, Arc is known to be required for the formation of long-term spatial and fear memories (Plath et al., 2006; Minatohara et al., 2015). Together, these data provide evidence that Chd1 functions in the same pathway as Egr1 and Arc to orchestrate downstream effects necessary for memory formation.

Expression of the transcription factor *Egr1* is regulated by input from various signaling cascades, including p38, MAPK or PI3K pathways (Veyrac et al., 2014; Duclot and Kabbaj, 2017). Temporally controlled binding of transcription factors activated by these pathways (e.g., ELK1, CREB, Sp1, Ap1) at the *Egr1* promoter, recruitment of cofactors, some of which modulate histone methylation, acetylation and phosphorylation patterns at promoter nucleosomes, and dynamic nucleosome positioning have been shown to result in stimulus-dependent activation of the gene (Tur et al., 2010; Riffo-Campos et al., 2015). In turn, Egr1 protein is able to bind to its own promoter upon nucleosome remodeling. Interaction of Egr1 with co-repressors, such as Nab1 and Nab2, and potentially the nucleosome remodeling and deacetylase complex NuRD, causes the subsequent downregulation of its own transcription (Tur et al., 2010; Riffo-Campos et al., 2015; Duclot and Kabbaj, 2017). In light of the finding that *Egr1* transcription regulation involves the repositioning of nucleosomes at its promoter (Riffo-Campos et al., 2015), a possible explanation for the observed dysregulation of *Egr1* in the absence of a fully functional Chd1 is that Chd1 is directly involved in nucleosome remodeling at the *Egr1* promoter. Indeed, our data show that Chd1 becomes enriched in the promoter region of *Egr1* upon stimulation of transcription. Potentially, truncated Chd1 protein expressed in the mutant animals might no longer be able to stably associate with the promoter or, alternatively, might not be able to interact *via* its N-terminus with critical promoter-bound co-regulators resulting in insufficient remodeling and, consequently, in a lack of transcriptional activation. This idea is consistent with the observed decreased basal levels of *Egr1* and *Arc* in the HC of naïve *Chd1*^Δ2/Δ2^ compared to control *Chd1*^flox/flox^ mice. By contrast, stimulation by exposure to a new environment caused significantly increased *Egr1* levels in mutant compared to wild-type dHC indicating that activation of *Egr1* transcription in response to a stimulus is not fully dependent on Chd1. Moreover, because Chd1 is known to also function in transcription elongation by reassembling nucleosomes in the wake of RNA polymerase II (Skene et al., 2014), enhanced *Egr1* transcript levels in *Chd1*-mutant HC may be due in part to inappropriate reconstitution of the transcribed chromatin, which in turn increases transcriptional output by promoting RNA polymerase II elongation efficiency.

The observed overactivation in response to a new environment in *Chd1*^Δ2/Δ2^ mice on one hand, and the underactivation in the situation of deficient long-term memory retrieval on the other hand provide support for a model in which Chd1 and in particular its N-terminal phosphorylatable domain may be required for fine-tuning the extent of transcription at the *Egr1* gene locus in a way that can be modulated by the type of stimulatory input.

It is known for many genes that regulation is achieved by opposing actions of activating and repressive chromatin regulatory forces (e.g., Morris et al., 2014; de Dieuleveult et al., 2016). Loss of fully functional Chd1 might tip the balance in favor of repressive chromatin remodeling by, for instance, the NuRD chromatin remodeling complex. While at this point the regulatory activity of NuRD at the *Erg1* promoter is inferred from the ability of Nab1/2 to associate with NuRD (Duclot and Kabbaj, 2017), it is tempting to speculate that an imbalance of activating and repressive chromatin remodeling activities may alter transcriptional programs upon neuronal stimulation and thereby interfere with higher order processes, such as learning and memory.

In this study we showed for the first time that the chromatin remodeler Chd1 is involved in the regulation of memory, in particular, spatial memory, and we identified the N-terminal serine-rich region as a critical domain in this process. An interesting question for future studies will be to elucidate the exact functional position of Chd1 in the complex interplay of factors regulating the activity of learning and memory genes, such as *Egr1* and *Arc*.

## Author Contributions

IS, AM, AS, CS and AL conceived the study and designed the experiments. IS, AM, AS, AW FG-V, PP, JJL, MK and AL performed the experiments. IS, AM, AS, AW, LT, GL-R, JJL, NS, CS and AL analyzed the data and wrote the manuscript.

## Conflict of Interest Statement

The authors declare that the research was conducted in the absence of any commercial or financial relationships that could be construed as a potential conflict of interest.
