# Supplementary material for: Role for Chromatin Remodeling Factor Chd1 in Learning and Memory
Source: Front Mol Neurosci. 2019 Jan 23;12:3. doi: 10.3389/fnmol.2019.00003 (PMC6351481; doi:10.3389/fnmol.2019.00003)
Supplement: Supplementary file 1 [file Data_Sheet_1.pdf]

Supplementary Material

Role for chromatin remodeling factor Chd1 in learning and memory

Ines Schoberleitner[1§], Anna Mutti[2§], Anupam Sah[3§], Alexandra Wille[1], Francisco Gimeno-Valiente[4], Paolo Piatti[1,5], Maria Kharitonova[3], Luis Torres[4], Gerardo López-Rodas[4], Jeffrey J. Liu[6], Nicolas Singewald[3], Christoph Schwarzer[2], Alexandra Lusser[1*]

Correspondence

Alexandra Lusser

alexandra.lusser@i-med.ac.at

Supplementary Figure S1

Supplementary Figure S2

Supplementary Figure S3

Full Scan of western blots

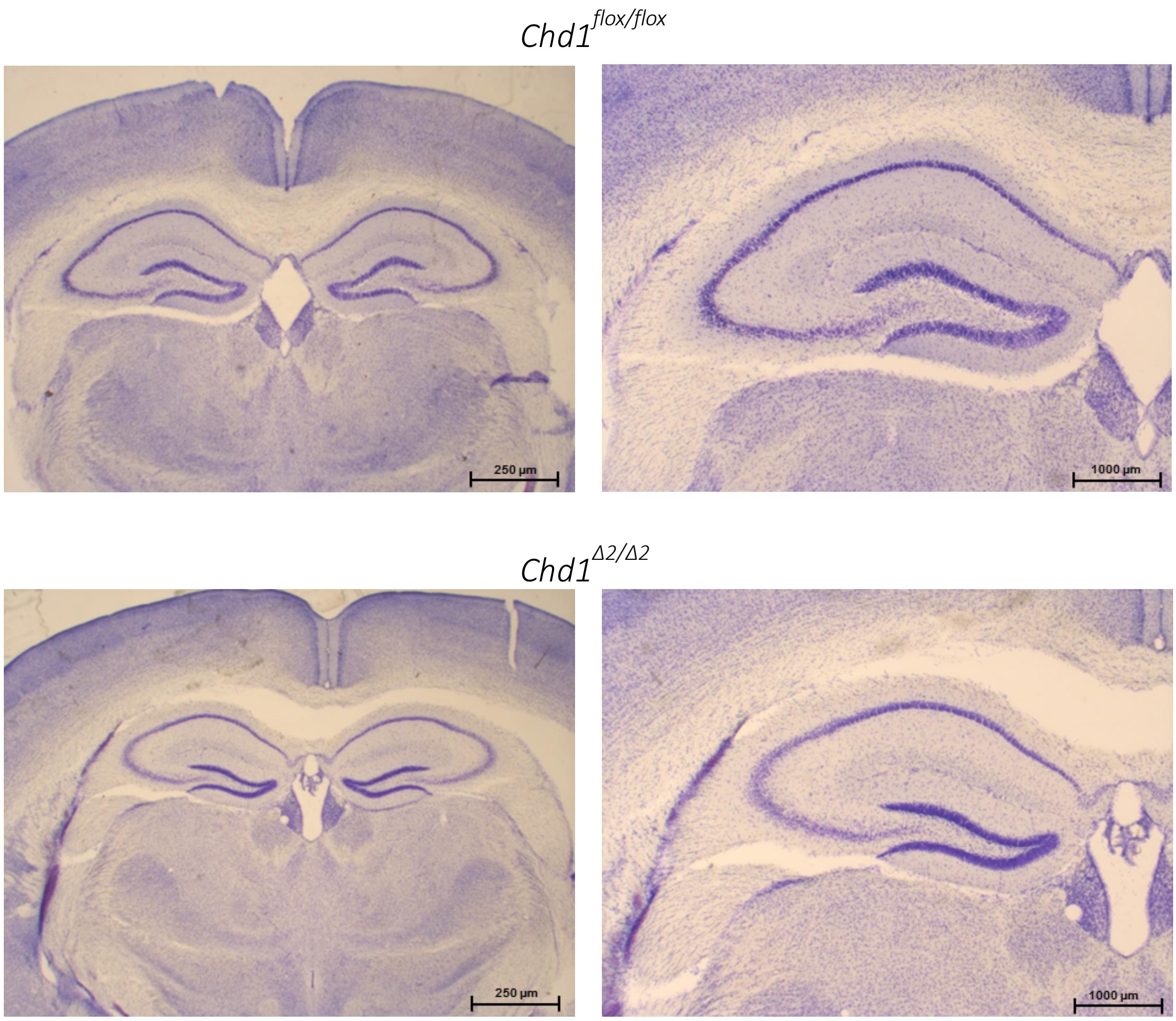

Suppl. Fig. S1 Nissl staining of dorsal hippocampus from representative *Chd1*$^{flox/flox}$ (top) and *Chd1*$^{\Delta2/\Delta2}$ (bottom) mice. The images on the right are magnifications of the left hippocampi from the same animals. Brains from n=5 mice/group were perfused with 4% PFA, embedded into gelatin and cut with a vibratome into 40 µm sections. A series of 6-10 sections containing dorsal and part of the ventral hippocampi were subjected to Nissl staining. No differences in overall anatomical structure of the dorsal hippocampi were detected.

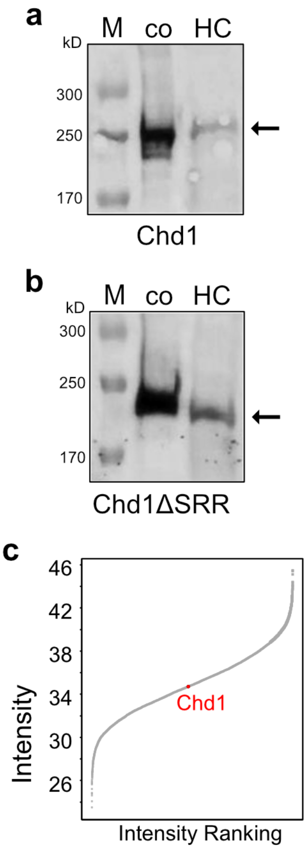

Suppl. Fig. S2 Expression of Chd1 in the mouse hippocampus. **a, b** Western blot with hippocampal nuclear protein extracts from *Chd1*$^{flox/flox}$ (**a**) and *Chd1*$^{\Delta 2/\Delta 2}$ (**b**) mice. *co*, an aliquot of recombinant full length Chd1 (**a**) as well as recombinant Chd1ΔSRR (**b**) was loaded as a size reference. *M*, size marker, *HC*, hippocampus. **c** Results from a quantitative mass spectrometry analysis of hippocampal protein extracts show Chd1 as a protein with average abundance.

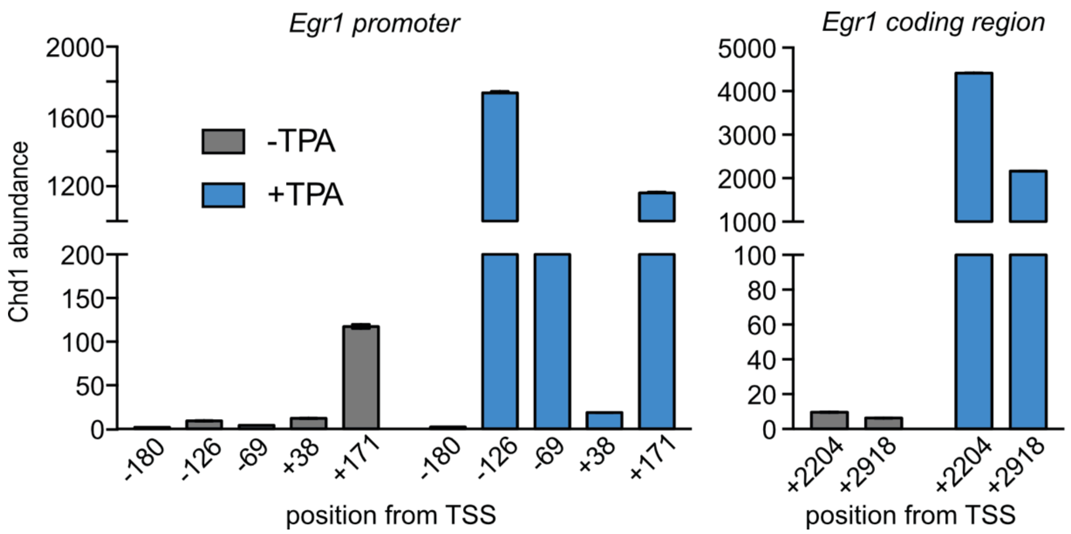

Suppl. Fig. S3 qPCR signals of Chd1 ChIP reactions normalized against non-antibody control reactions are shown for the non-induced (grey bars) and induced (by TPA addition) states (blue bars) of the *Egr1* gene. Mean ± SD of three (left graph) and two (right graph) technical replicates are shown.

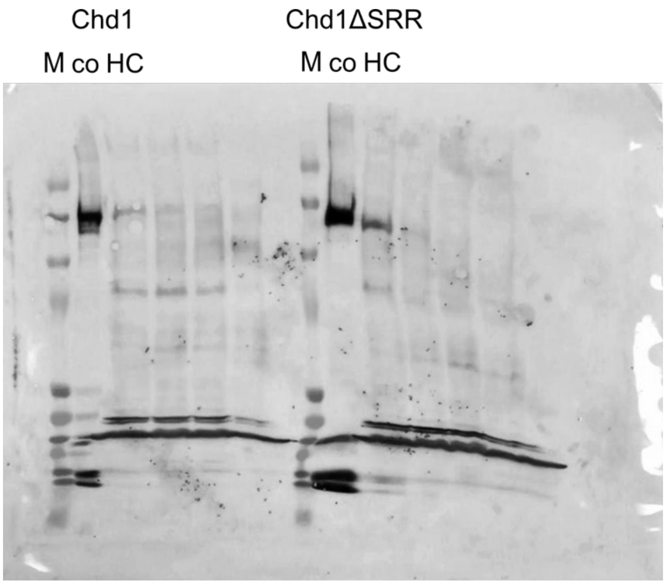

Full Scan of Western blot from Supplementary Figure S2.
